# Intermittent cold-blood vs. warm-blood cardioplegia – a comparative study on myocardial acidosis during coronary bypass surgery

**DOI:** 10.1186/1749-8090-8-S2-P2

**Published:** 2013-10-23

**Authors:** A Borowski, E Godehardt, G Paprotny, M Kurt

**Affiliations:** 1 Cardiovascular Surgery, University of Duesseldorf, Germany

## 

The aim of the study was to assess the impact of the intermittent cold-blood cardioplegia (ICBC) on myocardial acidosis in comparison to the warm-blood cardioplegic administration (IWBC) in patients undergoing coronary bypass surgery.

In 15 patients undergoing elective bypass surgery with ICBC for myocardial protection, metabolic changes of global ischemia indicators with lactate and pH values, measured simultaneously in the arterial and coronary sinus blood, were analyzed and compared with those observed when warm-blood method was used. For comparison, the analysis of variance with repeated measurements including tests for a cross-over effect of group and time was used.

In patients with ICBC compared to IWBC, the lactate production as well as degree of acidosis were generally lower with a significant group effect for both, lactate and pH values measured at the end of reperfusions. Our results suggest that ICBC has an inhibiting effect on potentially progressive myocardial acidosis during cross clamp period.

